# Gene co-expression networks in peripheral blood capture dimensional measures of emotional and behavioral problems from the Child Behavior Checklist (CBCL)

**DOI:** 10.1038/s41398-020-01007-w

**Published:** 2020-09-23

**Authors:** Jonathan L. Hess, Nicholas H. Nguyen, Jesse Suben, Ryan M. Meath, Avery B. Albert, Sarah Van Orman, Kristin M. Anders, Patricia J. Forken, Cheryl A. Roe, Thomas G. Schulze, Stephen V. Faraone, Stephen J. Glatt

**Affiliations:** 1 Department of Psychiatry & Behavioral Sciences, SUNY Upstate Medical University, Syracuse, NY USA; 2 Department of Psychology, Syracuse University, Syracuse, NY USA; 3 Department of Public Health and Preventive Medicine, SUNY Upstate Medical University, Syracuse, NY USA; 4 Institute of Psychiatric Phenomics and Genomics, Medical Center of the University of Munich, Munich, Germany; 5 Department of Psychiatry and Psychotherapy, University Medical Center Göttingen, Göttingen, Germany; 6 Department of Psychiatry and Behavioral Sciences, The Johns Hopkins University, Baltimore, MD USA; 7 Department of Genetic Epidemiology in Psychiatry, Central Institute of Mental Health, Mannheim, Germany; 8 Department of Neuroscience & Physiology, SUNY Upstate Medical University, Syracuse, NY USA

**Keywords:** Molecular neuroscience, Genomics, Human behaviour

## Abstract

The U.S. National Institute of Mental Health (NIMH) introduced the research domain criteria (RDoC) initiative to promote the integration of information across multiple units of analysis (i.e., brain circuits, physiology, behavior, self-reports) to better understand the basic dimensions of behavior and cognitive functioning underlying normal and abnormal mental conditions. Along those lines, this study examined the association between peripheral blood gene expression levels and emotional and behavioral problems in school-age children. Children were chosen from two age- and sex-matched groups: those with or without parental reports of any prior or current psychiatric diagnosis. RNA-sequencing was performed on whole blood from 96 probands aged 6–12 years who were medication-free at the time of assessment. Module eigengenes were derived using weighted gene co-expression network analysis (*WGCNA*). Associations were tested between module eigengene expression levels and eight syndrome scales from parent ratings on the Child Behavior Checklist (CBCL). Nine out of the 36 modules were significantly associated with at least one syndrome scale measured by the CBCL (i.e., aggression, social problems, attention problems, and/or thought problems) after accounting for covariates and correcting for multiple testing. Our study demonstrates that variation in peripheral blood gene expression relates to emotional and behavioral profiles in children. If replicated and validated, our results may help in identifying problem or at-risk behavior in pediatric populations, and in elucidating the biological pathways that modulate complex human behavior.

## Introduction

The prevailing diagnostic systems, such as the Diagnostic and Statistical Manual of Mental Disorders (DSM) and International Classification of Diseases (ICD), outline the criteria used for diagnosing psychiatric disorders. These criteria are not grounded on biological mechanisms. Psychiatric disorders have etiologies that are multifactorial, and many risk factors are known to be shared among disorders. The Research Domain Criteria (RDoC) framework initiated by the U.S. National Institute of Mental Health (NIMH) provides a multidimensional approach for studying psychiatric illness, and while not meant to immediately supplant the DSM, it provides a more flexible and quantitative framework for studying brain disorders and syndromes. The initiative explores fundamental levels of analysis for measurable cognitive, emotional, and behavioral constructs within discrete domains of functioning. RDoC units of analysis exist on a spectrum from essentially static units, such as DNA sequence, to dynamic units, such as behavioral phenomena. They also include intermediate molecular, neural, and physiological elements. The RDoC approach seeks to create a more valid or accurate representation of psychopathology and its underlying bases^1^.

A well-established tool for the dimensional assessment of childhood psychopathology is the Child Behavior Checklist (CBCL). Prior twin studies show that genetic influences explain a substantial portion of variation in CBCL scales, with heritability ranging from 15–48% for syndrome scales among 12,310 7-year old Dutch twin-pairs, and 54–71% for DSM-oriented scales among 398 Italian twin-pairs ages 8–17 years^2,3^. Although there is evidence that the CBCL is significantly heritable, no individual genes have yet been unequivocally associated with the CBCL from prior genome-wide association studies (GWASs). In such case, it is challenging to formulate mechanistic hypotheses for experimental studies of childhood psychopathology. Three GWASs of the CBCL have been published to date, resulting in 23 loci reaching suggestive levels of association (*p* ≤ 1 × 10^−5^) but none yet achieving genome-wide significance, indicating that studies have had insufficient sample sizes. Mick et al.^4^ performed a GWAS of 341 children affected with ADHD and found four loci associated with the CBCL dysregulation profile (also called pediatric bipolar disorder (BD) scale) defined as the sum of three CBCL syndrome scales (anxiety/depression, aggression, and attention). Kim et al.^5^ found five loci associated with the DSM-oriented CBCL scale for pervasive developmental problems (PDPs) and two for total problems score in a GWAS of 316 children, Benke et al.^6^ meta-analyzed single-SNP results from three independent GWAS of the CBCL scale for internalizing problems measured in 4596 children, and found 12 loci associated at suggestive significance. In addition, Benke et al. examined the joint effect of all GWAS SNPs on internalizing problem scores through heritability analysis. One nominally significant estimate of heritability was uncovered attributing 41% of the variance in internalizing problem scores to the effects of all SNPs in the GWAS, showing that the CBCL-internalizing problem score has a significant polygenic component similar to DSM-based psychiatric disorders^6^. Nevertheless, this finding should be interpreted with caution until it is confirmed through independent replication considering that Benke et al. failed to reproduce the effect using a different methodology in the same sample.

Other studies sought to attribute variance in CBCL scales to polygenic effects associated with neuropsychiatric disorders estimated in independent samples. One study of 5,947 children from the Generation R Study found that polygenic risk scores based on GWAS of schizophrenia were significantly associated with higher CBCL scores for internalizing problems^7^. A second population-based study of 2,437 children found that polygenic risk scores for ADHD were significantly associated with attention problems measured by the CBCL. These findings indicate that there is genetic overlap between dimensional psychopathology in children with diagnosis of schizophrenia and ADHD. A caveat of these studies is that they had not attributed genetic overlap to any specific loci. In absence of adequate sample sizes and reproducible genome-wide significant findings, it remains uncertain from the existing GWASs of the CBCL which genes contribute to problem behaviors in children.

Evaluating transcriptome-wide profiles for gene expression patterns associated with CBCL measures may help identify biological clues regarding genes and pathways relevant to childhood psychopathology. As a review by Tylee et al., prior transcriptomic studies have reported moderate to high concordance of gene expression levels across peripheral blood and postmortem brain (Pearson’s *r* = 0.25 to 0.64), suggesting that peripheral blood may serve as a useful proxy for the biological changes manifesting in the brain^8^. In addition, transcriptomic studies can potentially complement GWAS, and findings that converge across of the two approaches may help provide deeper insight into complex disorders. The goal of the current study was to identify gene co-expression modules and biological pathways underlying dimensional psychopathology in children measured by the CBCL. It was envisaged that the RDoC approach might eventually foster measures to better predict and prevent mental illness before onset based on objective, validated, laboratory-based measures^9,10^. We provided the first transcriptomic evaluation of module eigengenes and pathways associated with CBCL scales in a cohort of children that showed a broad range of behavioral and psychiatric profiles at a relatively young age (6–12 years). The findings from our study may inform subsequent efforts to develop better statistical models to predict onset or severity of mental illness and may shed light on new avenues for treatment. Our findings could be incorporated with other RDoC studies to help build better conceptual frameworks of mental illness agnostic to rigid DSM categories. We used a hypothesis-free discovery approach in our primary analysis considering no reproducible genome-wide significant loci have been reported to date. However, we hypothesized that gene expression signatures of the CBCL would show association with BD, schizophrenia, depression, and post-traumatic stress disorder (PTSD) given that the CBCL is predictive of those disorders. The CBCL is also significantly predictive of ADHD, however, we did not have transcriptomic data to evaluate the overlap between CBCL and ADHD transcriptomic signatures.

## Methods

### Experimental design overview

This is a pilot study of peripheral blood RNA expression levels related to CBCL scores in 96 probands aged 6–12 years. Here, we took an agnostic approach regarding psychopathology by ascertaining families having a child with any psychiatric diagnosis and sampling non-psychiatric children from the community (and their families) as well, allowing us to investigate a wide range of possible child behavior profiles. These children were sub-sampled from 2,806 individuals (954 probands from 954 families) who had been recruited into our longitudinal study of reward-system genetics. Probands were selected from two groups: those with a parent report of any prior or current psychiatric diagnosis, and those without (i.e., community-ascertained typically developing children). RNAs were extracted from whole blood and were sequenced and processed using standard bioinformatics pipelines to provide cross-sectional profiles of transcriptome-wide gene expression levels which could then be statistically associated with CBCL scores.

### Subject recruitment

Participants were recruited from a variety of sources within the Syracuse, NY and surrounding areas, including the Child and Adolescent Psychiatry Clinic at SUNY Upstate Medical University and child psychiatrists and mental health clinicians working in private practice in the community, as well as from community events (local fairs, festivals, etc.). Children were excluded for any of the following: sensorimotor disabilities, a diagnosed neurological condition, a history of head injury with documented loss of consciousness lasting more than 10 minutes, an uncontrolled medical condition, an IQ below 80 as reported by parents, or an inability to understand the English language. Adopted children were also excluded as this was designed as a family-genetic study. Informed consent was obtained from all parents and assent was obtained from all children upon arrival for their study visit. Study visits were approximately three hours in length and involved the completion of a variety of computerized inventories and behavioral paradigms. All probands were free of all psychiatric medications for at least six months prior to study assessment, with the exception that stimulants were allowed until within 12 hours of assessment. All protocols were approved by the Institutional Review Board at SUNY Upstate Medical University.

### Child Behavioral Checklist

The CBCL is a well-validated parent-reported measure of children’s emotional and behavioral functioning^11,12^. The CBCL includes 113 items for which parents were asked to assign a score based on a three-level rating scale indicating how true each item was for their child (0 = ‘Not true’, 1 = ‘Somewhat or sometimes true’, 2 = ‘Very true or often true’). The CBCL provides *t*-scores (minimum 50) in relation to U.S. national age- and gender-norms. For the present analysis, *t*-scores were evaluated for eight syndrome scales (anxious/depressed, withdrawn/depressed, somatic complaints, social problems, thought problems, attention problems, rule-breaking behavior, aggressive behavior). *T*-scores for nine other scales framed around DSM nosology were not included in our analysis (affective problems, anxiety problems, somatic problems, attention-deficit/hyperactivity problems, oppositional defiant problems, conduct problems, sluggish cognitive tempo, obsessive-compulsive problems, post-traumatic stress problems), as we aimed to describe gene expression changes that were predictive of dimensional measures of emotional and behavioral functioning rather than diagnoses. Nevertheless, the DSM-oriented scales of the CBCL were found to be highly correlated with the syndrome scales of the CBCL (Supplementary Fig. 1).

### Blood sample collection

Whole-blood samples were obtained from subjects at the time of their study visit. Blood was collected by a licensed phlebotomist into a 16 × 100 mm (2.5 mL whole blood, 6.9 mL additive) PAXgene Blood RNA (BD/Qiagen) tube. All samples were incubated for two hours at room temperature and then transferred to a freezer at –20 °C.

### RNA extraction, library preparation, sequencing, and quantification

Detailed procedures for these steps are provided in the Supplementary Materials and Methods. In brief, we followed standard procedures provided in a commercial RNA library preparation kit to prepare samples of RNA from whole blood for sequencing on an Illumina HiSeq 2000. A standard bioinformatics pipeline was used to align reads to the reference human genome (hg19), quantify counts aligned to genes, and normalize gene counts to reduce unwanted variation. Normalized gene expression values are presented in Supplementary Fig. 2.

### Deconvolution of cell types in whole blood based on gene expression levels

The deconvolution method proposed by Abbas et al. and implemented in the *R* software *CellMix* (version 1.6) was used to estimate the abundance of leukocytes from whole-blood gene expression levels across all probands based on a fixed set of cell-type-specific signatures^13,14^.

### Surrogate variable analysis (SVA)

We used the SVA algorithm implemented in the *R* package *sva* (version 3.30.1) to estimate hidden artefacts in our RNA-sequencing data set that might confound our statistical analyses^15,16^. An iteratively re-weighted least-squares approach was used to empirically estimate hidden artefacts called “surrogate variables”. The number of surrogate variables was selected using the asymptotic conditional singular value decomposition method (or “leek” method)^17^.

### Identification of gene co-expression networks

We generated a weighted gene co-expression network from normalized RNA-sequencing data using the *R* package weighted gene co-expression network analysis (*WGCNA*), the same procedure used in our previous blood-based gene expression mega-analyses^18–20^. Detailed procedures are provided in the Supplementary Materials and Methods.

### Pathway analysis

We performed a statistical test for gene set enrichment across module eigengenes to identify significant associations with CBCL syndrome scales using annotated gene sets from the Molecular Signatures Database (MSigDB)^21^. Additional details of our gene set analysis are provided in the Supplementary Materials and Methods. Enrichment *p*-values were adjusted using the Benjamini-Hochberg false discovery rate (FDR) procedure to correct for the number of gene sets tested within each module with a significance threshold set at FDR*p* < 0.05.

### Predicting CBCL scores with polytranscript risk scores

We used our approach called “polytranscript risk scoring” (akin to polygenic risk scoring) to estimate the proportion of variance in CBCL scales explained by a single linear composite score that summarizes the expression level of numerous genes along with an estimate of their effect size on CBCL scales and neuropsychiatric diagnoses^20^. Details of our approach are provided in the Supplementary Materials and Methods.

## Results

### Sample demographics and technical variables

Table 1 describes demographic and technical variables after the removal of one sample that had an insufficient number of paired-end sequenced reads (children with psychiatric concerns *n* = 47, typically developing children *n* = 48). Ten children of the 47 with psychiatric concerns reported prior use of psychiatric medications, but all were free of medication at the time of study assessment. No outliers were detected when we analyzed transcriptomic profiles with principal components analysis (Supplementary Fig. 3). The two groups did not differ in age (*t*-value = −0.06, *p* < 0.95), gender (*χ*^2^ = 0.009, *p* = 0.92), self-reported race (*χ*^2^ < 0.001, *p* < 0.99), experimentally determined RNA quality (*t*-value = 0.82, *p* = 0.41), total number of sequenced reads (*t*-value = −1.83, *p* = 0.07), GC content of reads (*t*-value = −1.01, *p* = 0.32), or overall quality of reads on the PHRED scale (*t*-value = −0.41, *p* = 0.68). Over 3.2 billion paired-end reads were generated from an Illumina HiSeq 2500 system with about 16.8 million reads produced per sample. Approximately 91% of reads were successfully aligned to the hg19 reference genome with a total of 51,833 Ensembl-annotated genes showing at least one read count in one sample. After data normalization and removal of low-abundance genes, 14,318 unique genes were retained for analysis, of which 11,529 were known to be protein-coding (81%). There were no significant differences between male and female probands with respect to ratings on CBCL syndrome scales (Supplementary Table 1). However, as expected, affected probands showed significantly higher ratings than typically developing probands for all CBCL syndrome scales (*p*-values < 0.01) apart from somatic complaints (*p* = 0.25).

**Table 1:** Comparison of demographic and technical factors between cases and comparison subjects.

Variable	Community-ascertained typical developing children	Cases
	(*n* = 48)	(*n* = 47)
	Number (%)	
Males	22 (46%)	23 (49%)
Caucasian	24 (50%)	24 (51%)
Medicated	0 (0%)	10 (22%)
	Mean (standard deviation)	
Age	9.3 (2.15)	9.36 (2.2)
RIN	8.8 (0.42)	8.7 (0.56)
PHRED (base call quality for RNA reads)	35.9 (0.22)	35.9 (0.12)
No. of sequenced reads	15,454,578 (5,889,023)	18,203,003 (6,528,747)
Read counts	5,996,077 (214,185)	6,434,947 (3,199,557)

### Hidden sources of confounding variation

The estimated abundance of circulating leukocytes was similar across gender (male and female, *p* > 0.1) and affection status (*p* > 0.1). One significant surrogate variable was detected (referred to as SV1) after accounting for variation in gene expression explained by age, gender, RNA quality, and race.

### Gene co-expression modules associated with CBCL scales

**Fig. 1:**
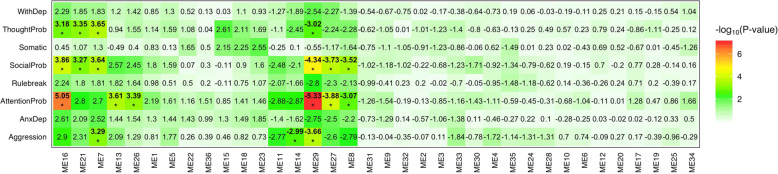
A heat-map showing associations between 36 module eigengenes (ME) and eight Child Behavior Checklist syndrome scales. Cell color refers to the significance of the association on the –log10(*p*-value) scale as denoted in the color legends in the left-hand margin. The signed values in the cells are *Z*-scores obtained from negative binomial regression. Hierarchical clustering with Ward’s algorithm was utilized to cluster columns according to pairwise similarity. Associations that remained significant after multiple-testing correction (Benjamini-Hochberg FDR*p* < 0.05) are bolded and marked with an asterisk (*). WithDep—Withdrawn/Depressed, ThoughtProb—Though problems, Somatic—Somatic complaints, SocialProb—Social problems, Rulebreak—Rule-Breaking Behavior, AttentionProb—Attention problems, AnxDep—Anxiety with depression.

The gene co-expression network obtained from *WGCNA* was divided into 36 modules that ranged in size from 41 genes to 2045 genes; 2601 genes that did not fit well with any module were pruned away from the network (Supplementary Fig. 4). Figure 1 shows all associations between CBCL scales and module eigengenes. After correcting for multiple comparisons, significant pairwise associations were detected for nine module eigengenes and four CBCL scales, namely: social problems (ME7, ME8, ME16, ME21, ME29, and ME27), thought problems (ME7, ME16, ME21, and ME29), attention problems (ME8, ME13, ME16, ME26, ME27, and ME29), and aggression (ME7, ME14, and ME29). Module eigengenes ME7, ME13, ME16, ME21, and ME26 showed positive associations with thought problems, social problems, attention problems, and/or aggression. Conversely, module eigengenes ME8, ME27, and ME29 displayed negative associations with social problems, attention problems, and/or aggression. The strongest association observed across all comparisons was between ME29 and attention problems (β = −5.33, SE = 0.15, *p* = 9.9 × 10^−8^).

Module eigengenes explained a relatively small proportion of variance in CBCL syndrome scales after accounting for the variance attributable to age, sex, race, RIN, and one surrogate variable (mean Nagelkerke’s *R*^2^ = 0.036, range: 0.0012–0.16). On average, module ME29 explained the largest amount of variance in CBCL syndrome scales (Nagelkerke’s *R*^2^ = 0.016). A mean Nagelkerke’s *R*^2^ = 0.036 was the amount of variance in each CBCL syndrome scale attributable to module eigengenes, on average, which ranged from 0.019 (withdrawn/depressed scale) to 0.065 (attention problems scale).

Significant associations between CBCL scales and individual genes from the nine significant *WGCNA* modules are presented in Supplementary Fig. 5. 1,209 gene transcripts out of 1,928 showed a significant association with at least one of the four CBCL scales after multiple-testing correction (FDR*p* < 0.05, total of 2,237 significant associations out of 4171 tests). We presented the top 20 most-strongly associated in Table 2, and provide summary statistics for all 2,237 significant gene-level associations in Supplementary File 1.

**Table 2:** A table with the four CBCL scales significantly associated with *WGCNA* modules, and the top 5 ranked genes associated with each scale. Genes are ordered from smallest to largest *p*-value.

CBCL scale	Module	Gene symbol	Beta	SE	*P*-value	FDRp
Aggression	ME7	*SLC9B2*	0.011	0.002	3.17 × 10^−5^	0.025
	ME7	*KIAA0101*	0.020	0.005	3.17 × 10^−4^	0.071
	ME7	*NDUFA4*	0.013	0.004	3.47 × 10^−4^	0.071
	ME7	*S100PBP*	0.014	0.004	3.70 × 10^−4^	0.071
	ME7	*NNT*	0.016	0.004	4.86 × 10^−4^	0.071
Attention problems	ME8	*PILRB*	−0.042	0.008	1.00 × 10^−6^	4.25 × 10^−4^
	ME8	*RP11-434B12.1*	−0.015	0.003	1.91 × 10^−6^	4.25 × 10^−4^
	ME16	*RNU6-4P*	0.015	0.003	1.94 × 10^−6^	4.25 × 10^−4^
	ME27	*RPUSD2*	−0.036	0.007	1.99 × 10^−6^	4.25 × 10^−4^
	ME8	*RP11-219E7.1*	−0.033	0.006	2.14 × 10^−6^	4.25 × 10^−4^
Social problems	ME8	*RP11-253E3.1*	−0.027	0.006	1.73 × 10^−5^	9.61 × 10^−3^
	ME8	*FAHD2CP*	−0.034	0.008	1.95 × 10^−5^	9.61 × 10^−3^
	ME8	*IGKV1-39*	−0.022	0.005	2.66 × 10^−5^	9.61 × 10^−3^
	ME8	*RP3-423B22.5*	−0.021	0.005	2.73 × 10^−5^	9.61 × 10^−3^
	ME16	*RNU6-4P*	0.015	0.003	4.11 × 10^−5^	0.01
Thought problems	ME7	*ADD1*	0.015	0.005	1.49 × 10^−3^	0.46
	ME7	*KIAA0101*	0.018	0.006	2.32 × 10^−3^	0.46
	ME29	*EOGT*	−0.015	0.005	2.87 × 10^−3^	0.46
	ME21	*ANKRD17*	0.011	0.004	3.58 × 10^−3^	0.46
	ME21	*DIABLO*	0.011	0.004	3.78 × 10^−3^	0.46

*ME* module eigengene, *SE* standard error, *FDRp* false discovery rate adjusted *p*-value.

### Gene set enrichment

No Gene Otology (GO) term showed a significant overlap with module eigengenes associated with CBCL scales after correction for multiple testing. The top twenty gene sets ranked by enrichment *p*-value are shown in Supplementary Fig. 6, which we categorized into four broad domains based on commonalities: regulation of DNA/transcription/chromatin, energy, immunity, and signaling.

### Polytranscript risk scores associated with CBCL scores

**Fig. 2:**
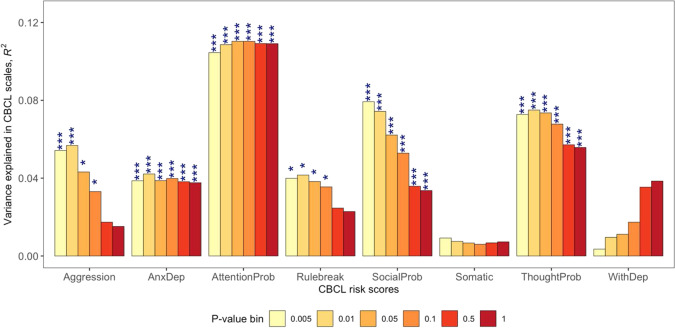
Average amount of variance in Child Behavior Checklist (CBCL) scales explained by CBCL polytranscript risk scores from 5-fold cross-validation. *P < 0.05, **P < 0.01, ***FDR-adjusted *p* < 0.05 (correcting for a total of six tests per CBCL scale).

Figure 2 shows the results from our five-fold cross-validation analysis of CBCL polytranscript risk scores. CBCL polytranscript risk scores explained a significant proportion of variance in five CBCL scales in the 20% of withheld samples from our five-fold cross-validation, namely: aggression (max *R*^2^ = 0.057, min *p* = 0.011, FDR*p* = 0.041), anxiety/depression (max *R*^2^ = 0.042, min *p* = 0.028, FDR*p* = 0.034), attention problems (max *R*^2^ = 0.11, min *p* = 0.00056, FDR*p* = 0.0008), social problems (max *R*^2^ = 0.079, min *p* = 0.004, FDR*p* = 0.016), and thought problems (max *R*^2^ = 0.075, min *p* = 0.0034, FDR*p* = 0.008).

**Fig. 3:**
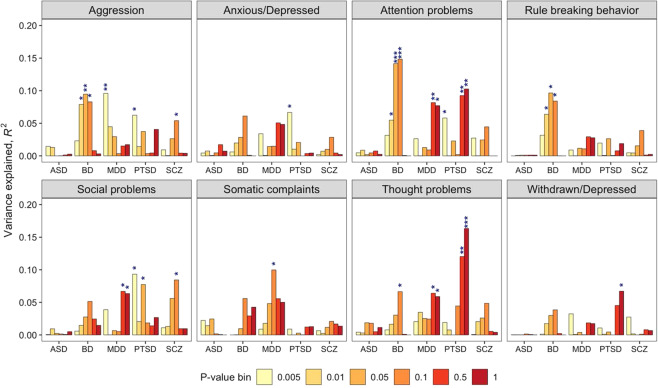
Association between Child Behavior Checklist syndrome scales and polytranscript risk scores for five psychiatric disorders. *P < 0.05, **P < 0.01, ***FDR-adjusted *p* < 0.05 (correcting for a total of 48 tests per disorder). autism spectrum disorder (ASD), bipolar disorder (BD), major depression (MDD), post-traumatic stress disorder (PTSD), schizophrenia (SCZ).

The average proportion of variance in each CBCL scale explained by polytranscript risk scores that were externally defined for psychiatric disorders in other samples is shown in Fig. 3. Three significant associations emerged after correction for multiple testing (FDR*p* < 0.05): polytranscript risk scores for BD were significantly associated with attention problems (*R*^2^ = 0.15, *p* = 0.0004), and polytranscript risk scores for PTSD were significantly associated with attention problems (*R*^2^ = 0.18, *p* = 0.0005) and thought problems (*R*^2^ = 0.11, *p* = 0.002).

## Discussion

We performed RNA-sequencing to profile transcriptome-wide gene expression in 95 children (1 sequencing outlier removed) to identify combinations of genes associated with emotional and behavioral problems measured by the CBCL using an agnostic sampling model^10^. We identified a gene network in our proband sample consisting of 36 gene co-expression modules. Nine modules (comprising 1,928 genes) showed a significant association with at least one of the following CBCL scales: aggression, thought problems, attention problems, and social problems. Biological pathways related to the regulation of DNA accessibility/transcription, metabolism, and immunity were implicated in our gene set enrichment analysis as potential drivers of the associations between module eigengenes and CBCL syndrome scales; however, our enrichment results are to be interpreted with caution as no single pathway achieved significance after multiple-testing correction. Although our findings ought to be interpreted with caution until replicated, the genes and pathways implicated in our study could serve as a basis for mechanistic hypotheses.

We cross-referenced all of our significant results against a brain-specific transcriptome-wide associations study of ADHD by Liao et al.^22^ to see whether any overlapping results existed. Expression of *MANBA*, a gene that encodes a lysosomal enzyme called mannosidase beta, was found in our study to be significantly positively associated with the CBCL attention problems scale and was found by Liao et al. to be significantly upregulated in the cerebellar hemisphere in ADHD^22^. A number of mutations in *MANBA* have been described that cause an extremely rare lysosomal storage disorder called beta-mannosidosis that typically presents with developmental delay and intellectual disability, and increased risk for seizures, infections, and hearing loss. Two of the approximately 20 reported cases with beta-mannosidosis presented with ADHD^23,24^, adding evidence in support of a potential etiological link between *MANBA* and ADHD. *MANBA* has also been implicated as a druggable gene and may offer a new avenue for ADHD treatment^25^.

Our study may be the first to look for transcriptomic markers of child psychopathology as characterized by several CBCL syndrome scales^10^. Measurement of the transcriptome in the neurons of living subjects would be ideal for examining the molecular biology of psychiatric illness. However, as this is not possible, we analyzed the peripheral blood as an accessible tissue that can also inform us of neurobiological correlates. As we previously reviewed in detail, existing studies comparing brain and blood transcriptomes suggest that 35–80% of known transcripts are expressed in both tissues and cross-tissue gene expression correlations ranged from 0.25 to 0.64^8^. Therefore, the transcriptomic dynamics in the blood can inform us of gene expression variability occurring in the brain, and may be able to illuminate biological mechanisms underlying mental illness.

Following on our findings, we quantified polytranscript risk scores for the CBCL scales and several psychiatric disorders, then assessed whether the polytranscript risk scores were able to serve as significant predictors of CBCL scores^20^. Our de novo polytranscript risk scores for CBCL scales showed a significant association with all CBCL scales except rule-breaking behavior, somatic complaints, and withdrawn/depressed scales. The three null findings are consistent with the lack of significant associations between these three scales and *WGCNA* modules. Blood-based gene expression levels do not exert strong effects on these specific scales. A follow-up study with larger sample sizes is warranted to determine if significant polytranscript risk scores for these three scales can be identified. Blood-based polytranscript risk scores (derived from independent samples) for BD and PTSD accounted for a significant proportion of the underlying transcriptomic correlates of the CBCL scales. This finding is consistent with prior work showing a shared molecular basis among psychiatric disorders in transcriptome-wide studies of postmortem brain tissues^26,27^, in addition to evidence of shared polygenic overlap between psychiatric disorders found by GWAS^28–31^. However, this is the first study to have evaluated the transcriptomic overlap between CBCL dimensions and psychiatric disorders. We found that polytranscript risk scores associated with BD accounted for a significant proportion of variance in scores for attention problems, while PTSD polytranscript risk scores were significantly associated with the CBCL thought problems scale. These findings indicate that BD and PTSD may each share a common set of genes and/or biological pathways with dimensional psychopathology in children. The attention problems scale has been shown to identify children with attention-deficit/hyperactivity disorder (ADHD)^32,33^, which is indirect evidence that transcriptomic signatures for dimensional scores for ADHD may overlap with BD. Elevated CBCL scores for thought problems have been associated with co-morbid mania in children with ADHD, implying a biological link between PTSD and ADHD that was supported by our study^34^. These findings fit with epidemiological studies that have shown heightened prevalence of BD and PTSD among individuals diagnosed with ADHD, as well as familial co-aggregation of these disorders^35–40^. It is unclear if the transcriptomic overlap we found is driven by shared genetic etiological factors, non-genetic factors, or a combination of the two.

Studying gene expression in the brains of living individuals is not feasible, thus we have used blood-based gene expression as a proxy for gene expression in the brain. Converging evidence suggests that some -omic profiles from brain are reflected in the blood^8,18,41–49^; thus, blood-based gene expression profiles can serve as a potentially informative proxy for the brain. Transcriptomic studies of peripheral blood may provide important information on immunologic pathways associated with psychopathology, or the activities of genes that mediate immune responses in the periphery but take on other roles in the central nervous system (c.f., *C4A*). In addition, peripheral blood can offer a window into environmental insults associated with increased risk for neurodevelopmental and neuropsychiatric disorders. In short, peripheral blood in humans has the potential to shed light on genes and biological pathways underlying psychopathology.

To the best of our knowledge, this is the first study that used a transcriptomic approach to identify genes and pathways associated with CBCL scales. Our results shed light on blood-based gene expression profiles associated with emotional and behavioral problems in children, as well as transcriptomic profiles of CBCL scales shared with mood and anxiety disorders. Our findings could be used to guide mechanistic studies to explore genes and pathways regulating normal behavioral functioning and mental illness.

## Supplementary information

Supplementary Figures

Supplementary Materials and Methods

Supplementary File_1

## Data Availability

Raw data are being submitted to RDoCdb for controlled access. Custom *R* scripts used for data analysis and full summary statistics can be shared upon request to the authors.

## Code availability

Custom written R scripts used to perform the analyses can be made available upon request to the corresponding author.

## References

[1] Sanislow CA (2010). Developing constructs for psychopathology research: research domain criteria. J. Abnorm. Psychol..

[2] Fedko, I. O. et al. Heritability of behavioral problems in 7-year olds based on shared and unique aspects of parental views. *Behav. Genet.*10.1007/s10519-016-9823-1 (2017). 10.1007/s10519-016-9823-1 PMC5306273 27796610

[3] Spatola, C. A. M. et al. A general population twin study of the CBCL/6-18 DSM-oriented scales. *J. Am. Acad. Child Adolesc. Psychiatry*10.1097/CHI.0b013e3180335b12 (2007). 10.1097/CHI.0b013e3180335b12 17450053

[4] Mick, E. et al. Genome-wide association study of the child behavior checklist dysregulation profile. *J. Am. Acad. Child Adolesc. Psychiatry*10.1016/j.jaac.2011.05.001 (2011). 10.1016/j.jaac.2011.05.001 PMC3143361 21784300

[5] Kim, D. S. et al. Results of genome-wide analyses on neurodevelopmental phenotypes at four-year follow-up following cardiac surgery in infancy. *PLoS ONE*10.1371/journal.pone.0045936 (2012). 10.1371/journal.pone.0045936 PMC3457986 23049896

[6] Benke, K. S. et al. A genome-wide association meta-analysis of preschool internalizing problems. *J. Am. Acad. Child Adolesc. Psychiatry* 2014, 10.1016/j.jaac.2013.12.028 (2014). 10.1016/j.jaac.2013.12.028 24839885

[7] Jansen, P. R. et al. Polygenic scores for schizophrenia and educational attainment are associated with behavioural problems in early childhood in the general population. *J. Child Psychol. Psychiatry Allied Discip.*10.1111/jcpp.12759 (2018). 10.1111/jcpp.12759 28627743

[8] Tylee DS, Kawaguchi DM, Glatt SJ (2013). On the outside, looking in: a review and evaluation of the comparability of blood and brain ‘-omes’. Am. J. Med. Genet. B Neuropsychiatr. Genet..

[9] Cuthbert, B. N. & Insel, T. R. Toward the future of psychiatric diagnosis: the seven pillars of RDoC. *BMC Med.*10.1186/1741-7015-11-126 (2013). 10.1186/1741-7015-11-126 PMC3653747 23672542

[10] Cuthbert, B. N. The RDoC framework: facilitating transition from ICD/DSM to dimensional approaches that integrate neuroscience and psychopathology. *World Psychiatry*10.1002/wps.20087.(2014). 10.1002/wps.20087 PMC3918011 24497240

[11] Achenbach TM (1991). Integrative Guide ot the 1991 CBCL/4-18 YSR, and TRF Profiles. Univ. Vt., Dep. Psychol. Pediatr..

[12] Wu JY (2001). Manual for the ASEBA school-age forms and profiles. J. Child Neurol..

[13] Abbas AR, Wolslegel K, Seshasayee D, Modrusan Z, Clark HF (2009). Deconvolution of blood microarray data identifies cellular activation patterns in systemic lupus erythematosus. PLoS ONE.

[14] Gaujoux R, Seoighe C (2013). CellMix: a comprehensive toolbox for gene expression deconvolution. Bioinformatics.

[15] Leek JT, Storey JD (2007). Capturing heterogeneity in gene expression studies by surrogate variable analysis. PLoS Genet..

[16] Leek, J. T. et al. sva: Surrogate Variable Analysis. R Packag. version 3.20.0. (2016).

[17] Leek JT (2011). Asymptotic conditional singular value decomposition for high-dimensional genomic data. Biometrics.

[18] Hess JL (2016). Transcriptome-wide mega-analyses reveal joint dysregulation of immunologic genes and transcription regulators in brain and blood in schizophrenia. Schizophr. Res..

[19] Tylee DS (2017). Blood transcriptomic comparison of individuals with and without autism spectrum disorder: a combined-samples mega-analysis. Am. J. Med Genet. Part B Neuropsychiatr. Genet..

[20] Hess, J. L. et al. Transcriptomic abnormalities in peripheral blood in bipolar disorder, and discrimination of the major psychoses. *Schizophr. Res*. 10.1016/j.schres.2019.07.036 (2019). 10.1016/j.schres.2019.07.036 PMC6997041 31391148

[21] Subramanian A (2005). Gene set enrichment analysis: a knowledge-based approach for interpreting genome-wide expression profiles. Proc. Natl Acad. Sci. USA.

[22] Liao C (2019). Transcriptome-wide association study of attention deficit hyperactivity disorder identifies associated genes and phenotypes. Nat. Commun..

[23] Sedel, F. et al. Atypical Gilles de la Tourette syndrome with β-mannosidase deficiency. *Arch. Neurol*. 10.1001/archneur.63.1.129. (2006). 10.1001/archneur.63.1.129 16401745

[24] Blomqvist, M. et al. β-Mannosidosis caused by a novel homozygous intragenic inverted duplication in MANBA. *Cold Spring Harb. Mol. Case Stud.*10.1101/mcs.a003954 (2019). 10.1101/mcs.a003954 PMC6549551 30886116

[25] Hegvik, T.-A. et al. Druggable genome in attention deficit/hyperactivity disorder and its co-morbid conditions. New avenues for treatment. *Mol. Psychiatry*10.1038/s41380-019-0540-z (2019). 10.1038/s41380-019-0540-z PMC7165040 31628418

[26] Gandal, M. J. et al. Shared molecular neuropathology across major psychiatric disorders parallels polygenic overlap. *Science*10.1126/science.aad6469 (2018). 10.1126/science.aad6469 PMC5898828 29439242

[27] Gandal, M. J. et al. Transcriptome-wide isoform-level dysregulation in ASD, schizophrenia, and bipolar disorder. *Science*10.1126/science.aat8127 (2018). 10.1126/science.aat8127 PMC6443102 30545856

[28] Zheng, J. et al. LD Hub: a centralized database and web interface to perform LD score regression that maximizes the potential of summary level GWAS data for SNP heritability and genetic correlation analysis. *Bioinformatics* 2016; btw613. 10.1093/bioinformatics/btw613 PMC5542030 27663502

[29] Bulik-Sullivan B (2015). An atlas of genetic correlations across human diseases and traits. Nat. Genet..

[30] Lee, S. H. et al. Genetic relationship between five psychiatric disorders estimated from genome-wide SNPs. *Nat. Genet*. 10.1038/ng.2711 (2013). 10.1038/ng.2711 PMC3800159 23933821

[31] Lee, P. H. et al. Genomic relationships, novel loci, and pleiotropic mechanisms across eight psychiatric disorders. Cell 10.1016/j.cell.2019.11.020 (2019). 10.1016/j.cell.2019.11.020 PMC7077032 31835028

[32] Kim, J. W. et al. The child behavior checklist together with the ADHD rating scale can diagnose ADHD in Korean community-based samples. *Can. J. Psychiatry*10.1177/070674370505001210 (2005). 10.1177/070674370505001210 16408529

[33] Lampert, T. L., Polanczyk, G., Tramontina, S., Mardini. V. & Rohde, L. A. Diagnostic performance of the CBCL-attention problem scale as a screening measure in a sample of Brazilian children with ADHD. *J. Atten. Disord.*10.1177/108705470400800204 (2004). 10.1177/108705470400800204 15801336

[34] Hazell, P. L., Lewin, T. J. & Carr, V. J. Confirmation that Child Behavior Checklist clinical scales discriminate juvenile mania from attention deficit hyperactivity disorder. *J. Paediatr. Child Health*10.1046/j.1440-1754.1999.t01-1-00347.x (1999). 10.1046/j.1440-1754.1999.t01-1-00347.x 10365361

[35] Sanchez-Gistau, V. et al. Psychiatric disorders in child and adolescent offspring of patients with schizophrenia and bipolar disorder: a controlled study. *Schizophr. Res.*10.1016/j.schres.2015.08.034 (2015). 10.1016/j.schres.2015.08.034 26363969

[36] Wang, L. J. et al. Attention-deficit hyperactivity disorder, its pharmacotherapy, and the risk of developing bipolar disorder: a nationwide population-based study in Taiwan. *J. Psychiatr. Res.*10.1016/j.jpsychires.2015.10.014 (2016). 10.1016/j.jpsychires.2015.10.014 26519764

[37] McIntyre, R. S. et al. Attention-deficit/hyperactivity disorder in adults with bipolar disorder or major depressive disorder: results from the international mood disorders collaborative project. *Prim. Care Companion J. Clin. Psychiatry*10.4088/PCC.09m00861gry (2010). 10.4088/PCC.09m00861gry PMC2947541 20944770

[38] Spencer, A. E. et al. Examining the association between posttraumatic stress disorder and attention-deficit/hyperactivity disorder: a systematic review and meta-analysis. *J. Clin. Psychiatry*10.4088/JCP.14r09479 (2016). 10.4088/JCP.14r09479 26114394

[39] Wozniak J (2019). Comorbidity of bipolar I disorder and conduct disorder: a familial risk analysis. Acta Psychiatr. Scand..

[40] van Hulzen KJE (2017). Genetic overlap between attention-deficit/hyperactivity disorder and bipolar disorder: evidence from genome-wide association study meta-analysis. Biol. Psychiatry.

[41] Sullivan PF, Fan C, Perou CM (2006). Evaluating the comparability of gene expression in blood and brain. Am. J. Med. Genet. Part B Neuropsychiatr. Genet..

[42] McKenzie M, Henders AK, Caracella A, Wray NR, Powell JE (2014). Overlap of expression Quantitative Trait Loci (eQTL) in human brain and blood. BMC Med. Genomics.

[43] Qi, T. et al. Identifying gene targets for brain-related traits using transcriptomic and methylomic data from blood. *Nat. Commun.*10.1038/s41467-018-04558-1 (2018). 10.1038/s41467-018-04558-1 PMC5995828 29891976

[44] Kundakovic, M. & Jaric, I. The epigenetic link between prenatal adverse environments and neurodevelopmental disorders. *Genes*10.3390/genes8030104 (2017). 10.3390/genes8030104 PMC5368708 28335457

[45] Aberg, K. A. et al. Methylome-Wide Association Study of Schizophrenia. *JAMA Psychiatry*10.1001/jamapsychiatry.2013.3730 (2014). 10.1001/jamapsychiatry.2013.3730 PMC4331014 24402055

[46] Yehuda, R. & Lehrner, A. Intergenerational transmission of trauma effects: putative role of epigenetic mechanisms. *World Psychiatry*10.1002/wps.20568 (2018). 10.1002/wps.20568 PMC6127768 30192087

[47] Houtepen, L. C. et al. Genome-wide DNA methylation levels and altered cortisol stress reactivity following childhood trauma in humans. *Nat. Commun.*10.1038/ncomms10967 (2016) 10.1038/ncomms10967 PMC4802173 26997371

[48] Han, L. K. M. et al. Epigenetic aging in major depressive disorder. *Am. J. Psychiatry*10.1176/appi.ajp.2018.17060595 (2018). 10.1176/appi.ajp.2018.17060595 PMC6094380 29656664

[49] Zannas, A. S., Provençal, N. & Binder E. B. Epigenetics of posttraumatic stress disorder: current evidence, challenges, and future directions. *Biol. Psychiatry*10.1016/j.biopsych.2015.04.003 (2015). 10.1016/j.biopsych.2015.04.003 25979620

