# Supplementary material for: Gene co-expression networks in peripheral blood capture dimensional measures of emotional and behavioral problems from the Child Behavior Checklist (CBCL)
Source: Transl Psychiatry. 2020 Sep 23;10:328. doi: 10.1038/s41398-020-01007-w (PMC7511314; doi:10.1038/s41398-020-01007-w)
Supplement: Supplementary file 1 — Supplementary Figures [file 41398_2020_1007_MOESM1_ESM.docx]

**Supplementary Tables and Figures**

Table of Contents

[Supplementary Table 1 2](#_Toc27941981)

[Supplementary Figure 1 3](#_Toc27941982)

[Supplementary Figure 2 4](#_Toc27941983)

[Supplementary Figure 3 5](#_Toc27941984)

[Supplementary Figure 4 6](#_Toc27941985)

[Supplementary Figure 5 7](#_Toc27941986)

[Supplementary Figure 6 8](#_Toc27941987)

Supplementary Table 1. Average scores for eight Child Behavior Checklist (CBCL) syndrome scale across 95 probands grouped by gender and affection status.

|  |  |  | Female (n = 50) | | Male (n = 45) | |  | Typical developing (n = 48) | | Cases (n = 47) | |  |
| --- | --- | --- | --- | --- | --- | --- | --- | --- | --- | --- | --- | --- |
| **CBCL syndrome scales** | **Mean** | **SD** | **Mean** | **SD** | **Mean** | **SD** | ***p*-value** | **Mean** | **SD** | **Mean** | **SD** | ***p*-value** |
| Anxious/depressed | 105.36 | 7.11 | 106.04 | 8.37 | 104.60 | 5.36 | 0.32 | 102.58 | 3.87 | 108.19 | 8.46 | 1.04×10^-04^ |
| Withdrawn/depressed | 106.92 | 7.74 | 106.86 | 8.63 | 106.98 | 6.71 | 0.94 | 104.81 | 5.86 | 109.06 | 8.83 | 7.21×10^-03^ |
| Somatic complaints | 104.31 | 6.11 | 104.66 | 6.80 | 103.91 | 5.30 | 0.55 | 103.58 | 5.75 | 105.04 | 6.44 | 0.25 |
| Social problems | 107.73 | 8.20 | 108.26 | 9.40 | 107.13 | 6.67 | 0.50 | 104.35 | 5.94 | 111.17 | 8.79 | 3.07×10^-05^ |
| Thought problems | 108.11 | 8.09 | 107.98 | 8.73 | 108.24 | 7.42 | 0.87 | 104.85 | 5.66 | 111.43 | 8.88 | 5.06×10^-05^ |
| Attention problems | 109.11 | 9.17 | 109.48 | 10.67 | 108.69 | 7.24 | 0.67 | 104.02 | 5.06 | 114.30 | 9.54 | 8.75×10^-09^ |
| Rule-breaking behavior | 106.88 | 7.47 | 107.28 | 7.80 | 106.44 | 7.14 | 0.59 | 104.60 | 6.82 | 109.21 | 7.45 | 2.24×10^-03^ |
| Aggression | 107.34 | 8.60 | 108.12 | 9.94 | 106.47 | 6.82 | 0.34 | 103.94 | 5.42 | 110.81 | 9.84 | 7.50×10^-05^ |

SD – standard deviation, CBCL – Child Behavior Checklist. “Cases” refer to children with a parent report of a psychiatric diagnosis.


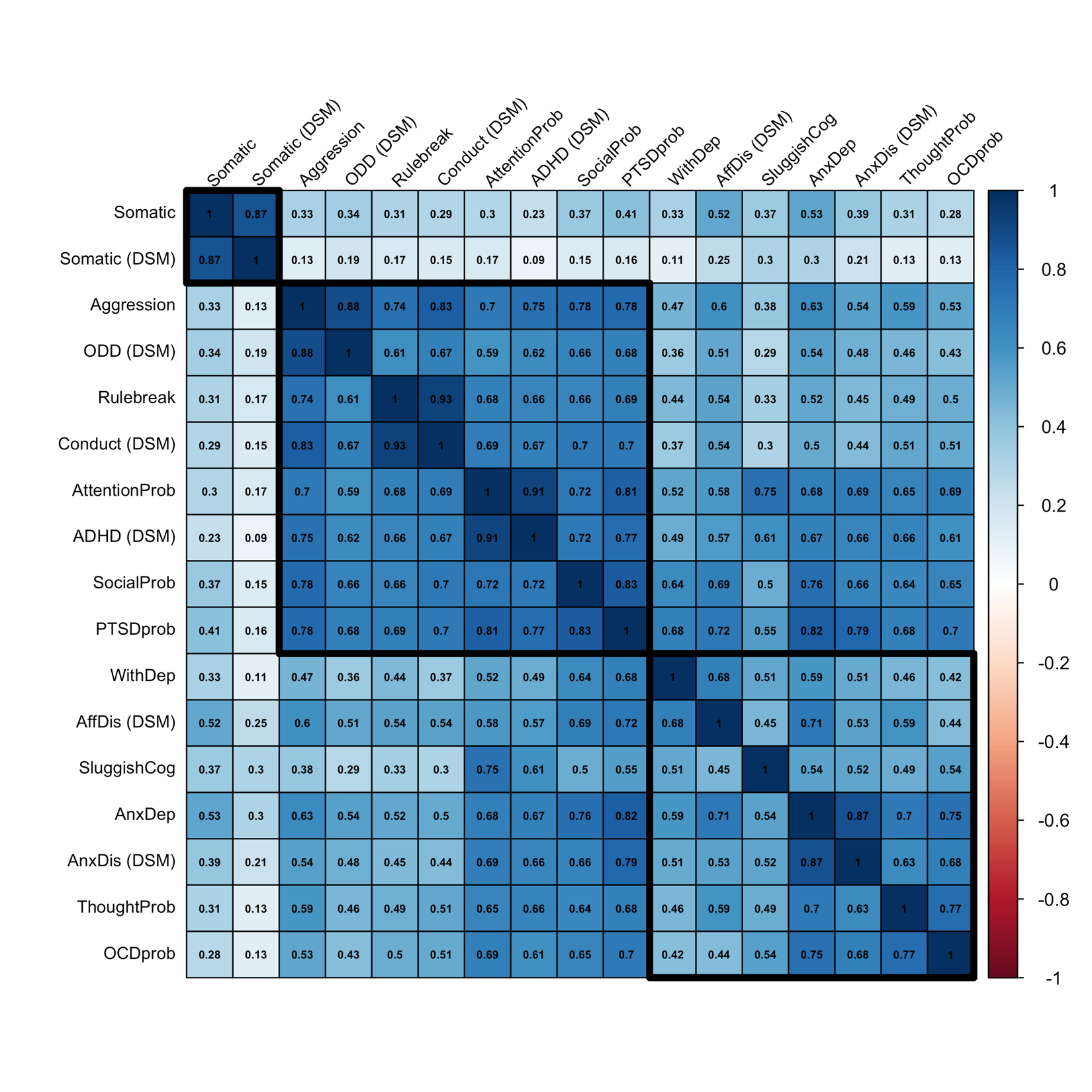


Supplementary Figure 1. Pair-wise Pearson’s correlation coefficients estimated between Child Behavior Checklist (CBCL) scales. Rows and columns were ordered using hierarchical clustering based on Euclidean distances. Boxes were drawn around three distinct clusters.

**
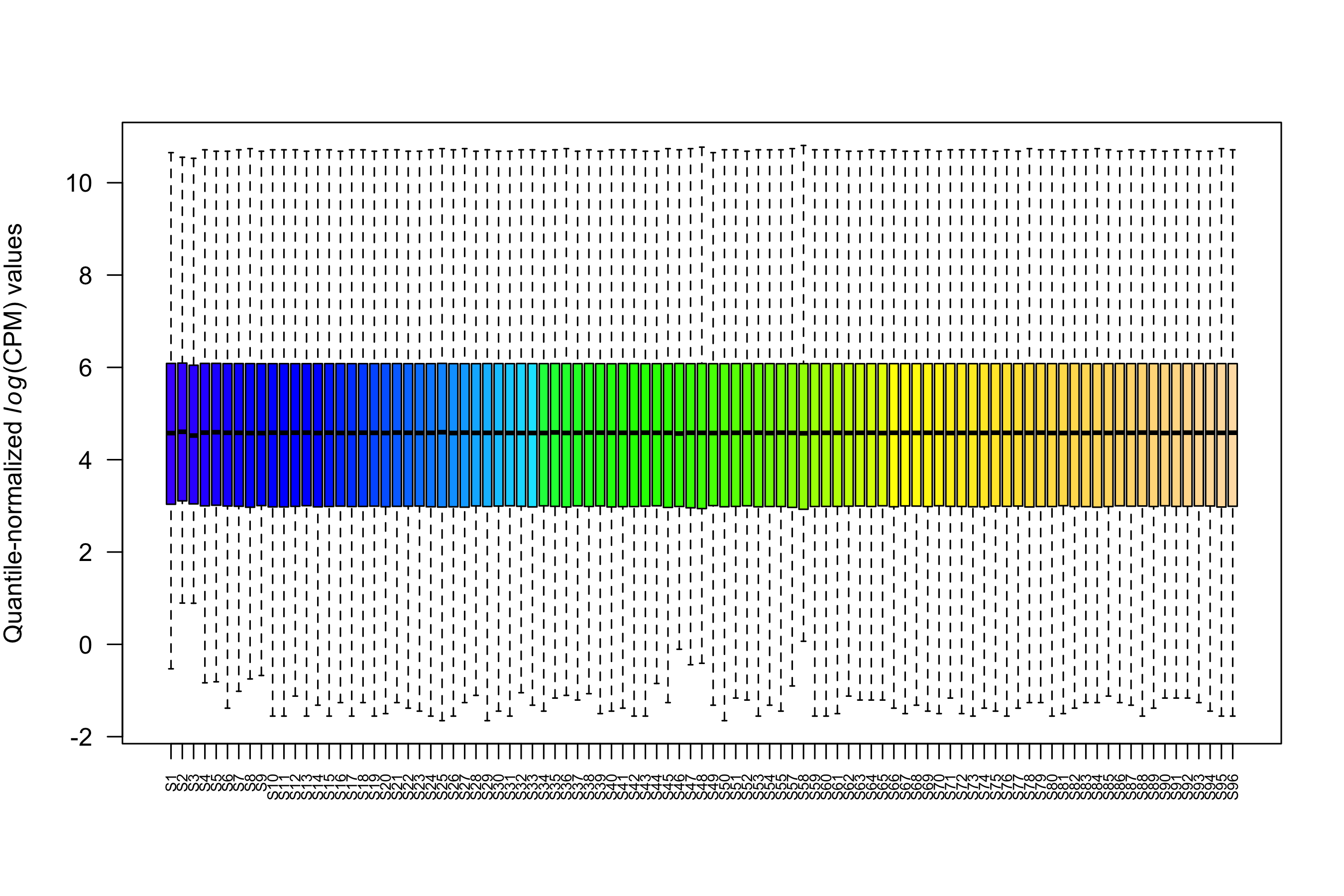
**

Supplementary Figure 2**.** Box-and-whisker plots showing normalized RNA-sequencing read count distributions for 95 samples (excluded one sample with an insufficient number of reads).

**
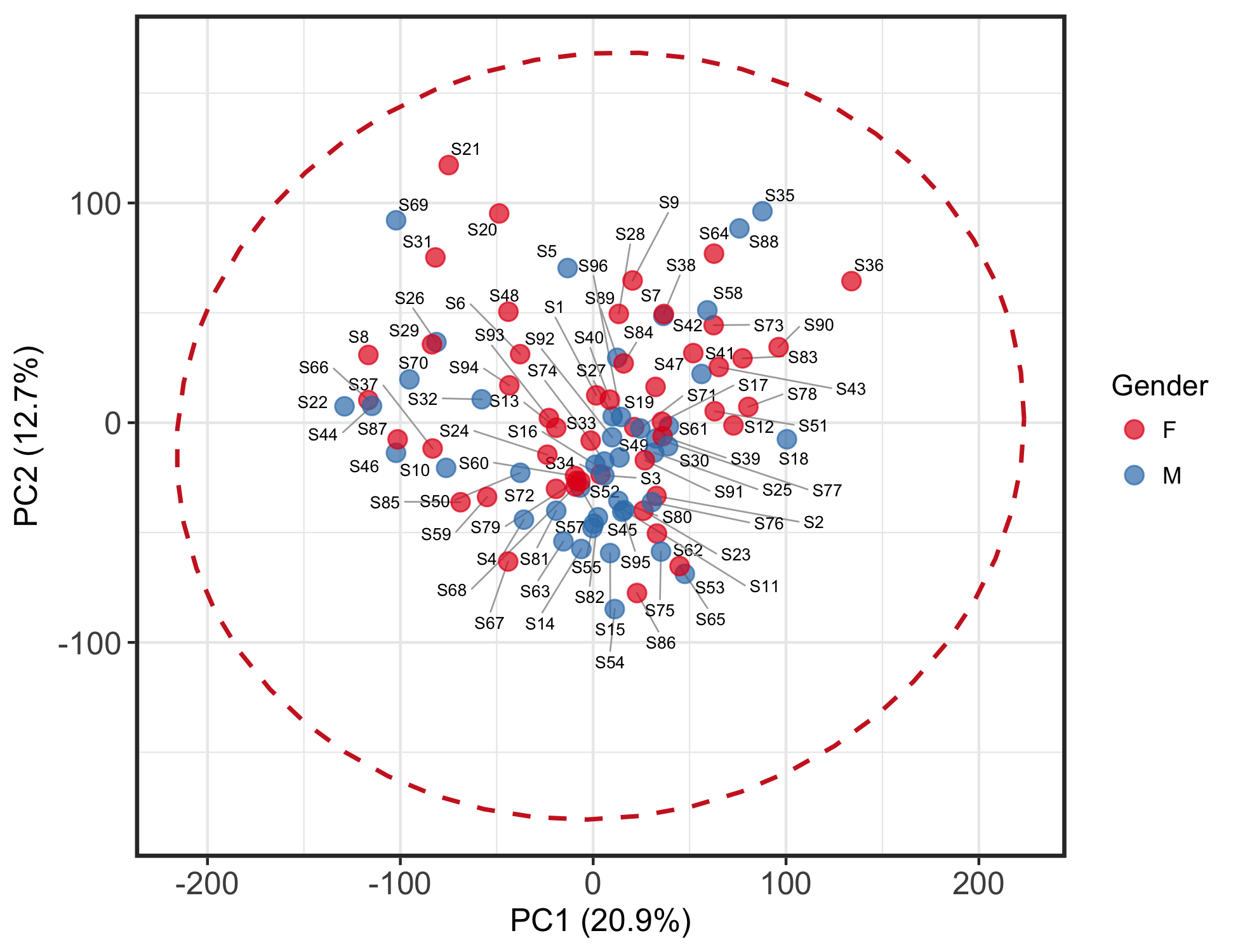
**

Supplementary Figure 3**.** Scatterplot of first two components from principal component analysis supplied with normalized read counts from 14,318 genes from 95 samples. A red dashed ellipse was drawn 4 standard deviations out from the center of the group.


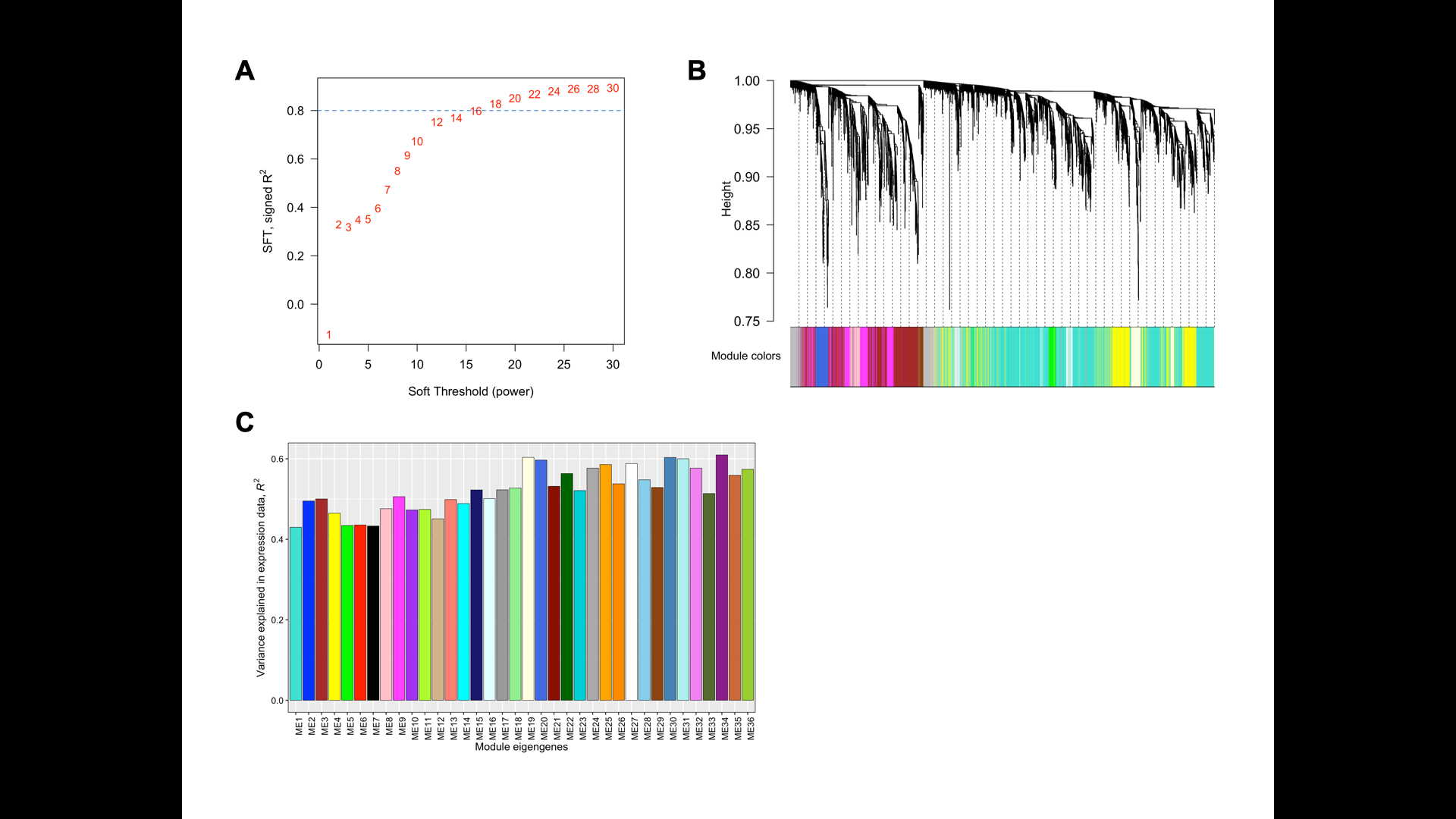


Supplementary Figure 4**.** (A) Twenty possible soft-threshold power options were evaluated. A power of 20 was selected as this approximated scale-free-topology for a signed network. (B) A dendrogram plot showing 36 gene co-expression modules that were detected from normalized RNA-sequencing reads counts in our sample of 95 probands. (C) Proportion of variance in gene expression levels captured by module eigengenes.

**
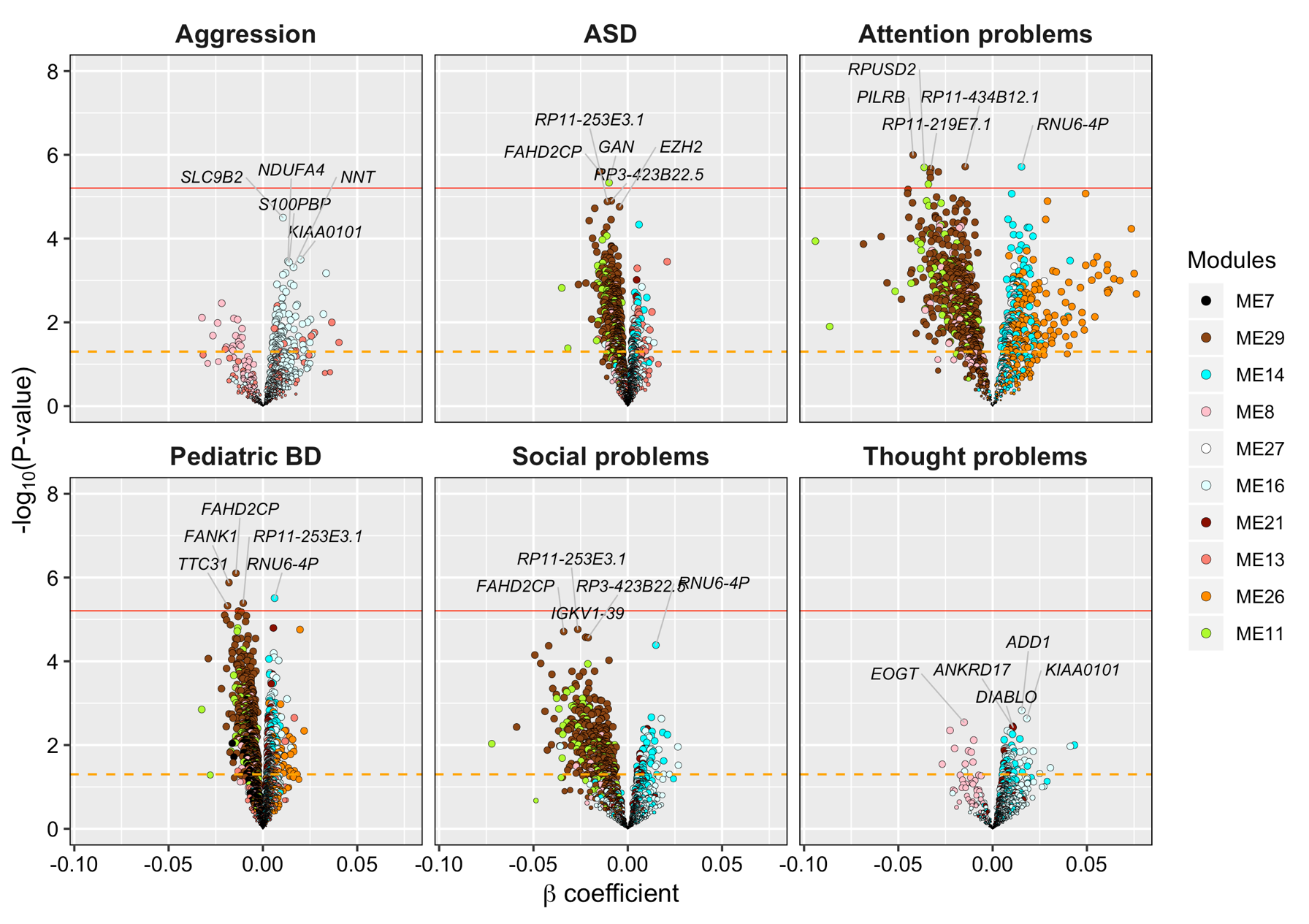
**

Supplementary Figure 5**.** Six volcano plots are presented which summarize the associations between six CBCL scales a total of 2,256 across 10 *WGCNA* modules found to be significantly associated with at least one of the six CBCL scales. The orange dotted line in each panel denotes a nominal significance threshold of p = 0.05, whereas the solid red line represents the Bonferroni-adjusted significance threshold of p = 6.22×10^-6^ which accounts for the total number of tests performed (No. of tests = 8,040). The five most significant genes associated with each CBCL scale are labeled.


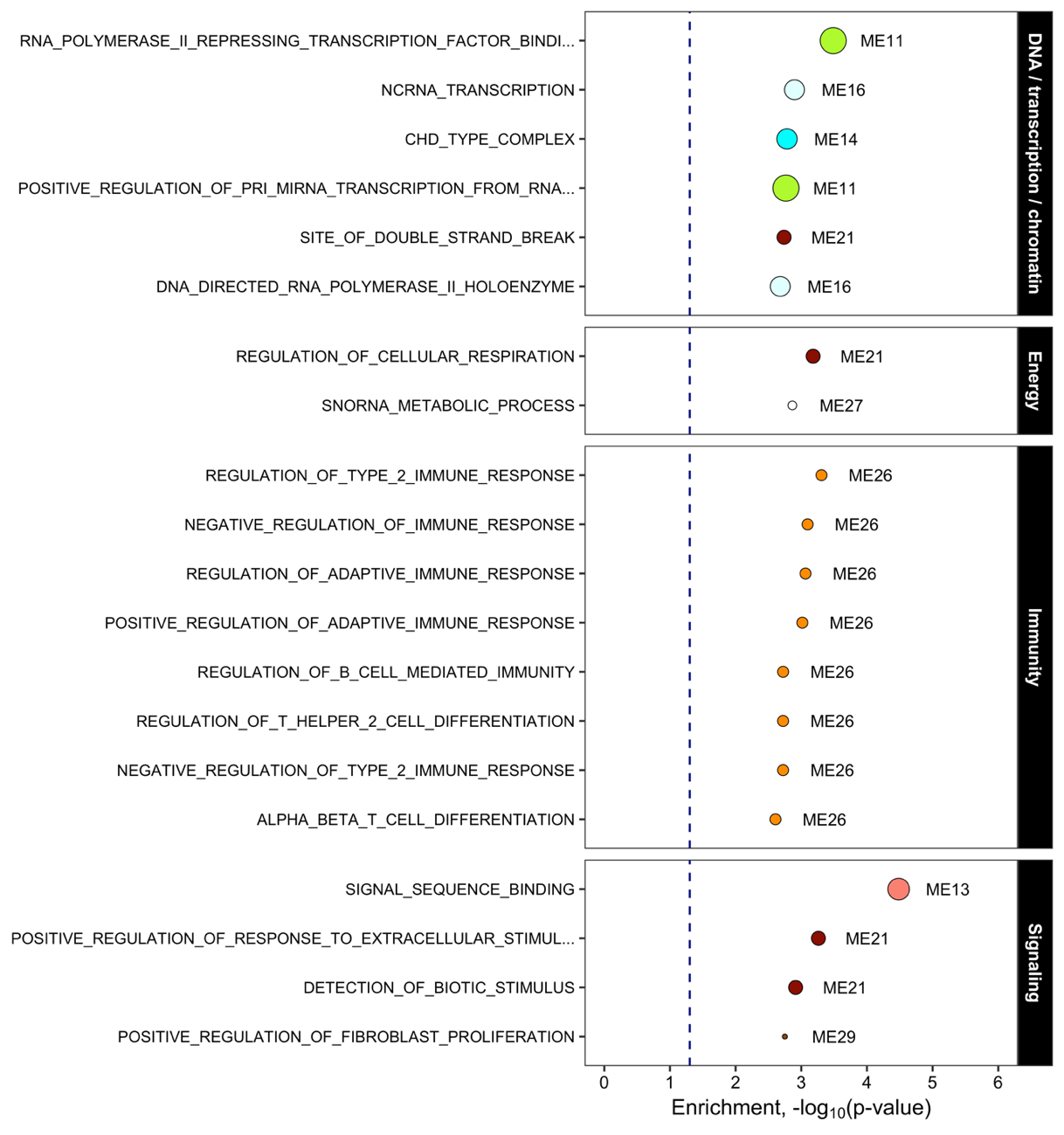


Supplementary Figure 6. The top 20 Gene Ontology (GO) terms from the Molecular Signatures Database that showed a nominally significant (*p*<0.05) enrichment among *WGCNA* module eigengenes that showed a significant association with CBCL scales. None of these gene sets survived multiple testing correction (Benjamin-Hochberg false-discovery rate adjustment). The size of each dot represents the number of genes contained in each module eigengene.
