# Supplementary material for: Gene co-expression networks in peripheral blood capture dimensional measures of emotional and behavioral problems from the Child Behavior Checklist (CBCL)
Source: Transl Psychiatry. 2020 Sep 23;10:328. doi: 10.1038/s41398-020-01007-w (PMC7511314; doi:10.1038/s41398-020-01007-w)
Supplement: Supplementary file 2 — Supplementary Materials and Methods [file 41398_2020_1007_MOESM2_ESM.docx]

**Supplementary Materials and Methods**

*Power analysis*

Since this is the first transcriptomic study of the CBCL, there is not a published set of effect sizes that we could use for a traditional power analysis estimating the probability of detecting a reliable effect of an anticipated magnitude. However, we performed a power analysis factoring in a range of hypothetical effect sizes (i.e., R^2^) ranging from a small to large proportion of variance explained in CBCL by module eigengenes and polytranscript risk scores (R^2^: 0.02 – 0.5). We estimated that our sample size (n=95) provided us with >80% power to detect an effect size of R^2^ = 0.09 at a significance threshold of p = 0.00018.

*RNA extraction, library preparation, and sequencing*

The RNA sample sequencing libraries were prepared at SUNY Upstate Medical University by RMM. RNA extraction was performed using PAXgene Blood RNA extraction kits (Qiagen) according to the kit protocol. RNA concentration and quality were determined by an Agilent Bioanalyzer. All samples used for further analysis had an RNA Integrity Number (RIN) higher than 8. Prior to library prep, samples were purified and concentrated to a final yield of 1µg in pure water using the RNA Clean and Concentrator 5 Kit (Zymo Research). Samples were prepared and pooled for RNA sequencing utilizing Illumina TruSeq Stranded Total RNA Library Prep kit with Ribo-Zero Globin following the kit’s protocol, which removes ribosomal RNA (cytoplasmic and mitochondrial) and globin messenger RNA. Samples were pooled into eight sets of 12. Samples were sequenced by 100 cycle paired-end reads on an Illumina HiSeq2500 at the UB Genomics and Bioinformatics Core (University at Buffalo, SUNY).

*Mapping and normalization of RNA-sequencing reads*

A quality control check was performed on raw sequencing reads contained in FASTQ formatted files using the software *FastQC* (version 0.11.8). Samples that had sufficient quantity (≥ 1 million) and quality of reads (mean PHRED ≥ 30) were retained for read mapping (*n* = 95). Read were aligned to hg19 version of the human reference genome with *STAR* *v2.5.0a*. Alignment was guided by annotated splice junctions in the Gencode v19 reference transcriptome assembly. Quantification of reads in genes was done with *featureCounts* (version 1.6.1) based on Ensembl gene annotations from the reference Gencode v19 GTF file (1, 2)*.* Quality control and data normalization were performed on read counts using *R*. Read counts were converted to counts per million (CPM) to adjust for library size and then were transformed to log_2_ values using *edgeR* (version 3.24.3) (3). Genes expressed at low abundance were discarded following criteria used by the CommonMind Consortium (≤ 1 CPM in < 50% of the sample) (4). Quantile normalization was applied to the log_2_CPM values to normalize gene expression values between samples (**Supplementary Figure 2**). A principal component analysis (PCA) was performed on normalized read counts to screen for potential outliers (none were detected) who were ≥4 standard deviations from the mean of all subjects for the first three principal components.

*Identification of gene co-expression networks*

A weighted gene co-expression network was derived using the *R* package *WGCNA* (version 1.67) (5)*.* The matrix of log_2_CPM values for 14,318 transcripts was supplied to the function *blockwiseModules* to perform automatic detection of a signed gene co-expression network based on pair-wise gene-gene correlations. The parameters used in the *blockwiseModules* function were as follows: *networkType = “signed”, TOMtype = “signed”, minModuleSize = 30, minCoreKMESize = 10, minKMEtoStay = 0, reassignThreshold = 1e-06, mergeCutHeight = 0.225, detectCutHeight = 0.995, corType = “bicor”.* This method computed a gene correlation matrix raised to the selected soft-threshold power of 20 which accentuated gene-pairs that were strongly correlated relative to gene-pairs with low correlation coefficients. An adjacency matrix was computed using the weighted correlation matrix between genes, which then was used to estimate topological similarity. Hierarchical clustering was performed on a dissimilarity matrix that was derived from topological similarities to detect gene modules (*i.e.,* groups of genes that were tightly co-expressed and share connections with other genes). Singular value decomposition was performed and the first principal component was extracted from each module (termed ‘module eigengene’) to summarize the overall expression profile of genes clustering in a module.

*Pathway analysis*

A total of 5,917 gene sets from Gene Ontology (GO) were downloaded from the Molecular Signatures Database (MSigDB version 6.2) (6). Gene sets that had less than 50% overlap with genes retained in our sample after quality control were discarded leaving 4,427 gene sets for enrichment analysis. A one-tailed hypergeometric test was used to determine if the overlap observed between gene sets and module eigengenes significantly associated with CBCL scores was greater than expected by chance. Gene sets sharing fewer than two genes with module eigengenes were excluded from enrichment tests (3,068 remaining gene sets). Enrichment scores (ES) were calculated using the following equation:$ES= \frac{m/n}{M/N}$ , wherein *m* is the number of genes shared by a module and gene set, *n* is the size of a module, *M* is the size of a gene set, and *N* is the number of genes measured across probands. Enrichment *p*-values were adjusted using the Benjamini-Hochberg false discovery rate (FDR) procedure to correct for the number of gene sets tested within each module with a significance threshold set at FDRp < 0.05.

*Predicting CBCL scores with polytranscript risk scores*

The polytranscript risk score is a weighted sum statistic, derived in an approach similar to that of polygenic risk scoring (7). Genes are first rank-ordered according to significance of association with CBCL syndrome scales using linear regression with adjustment for age, gender, RNA quality, and SV1. Genes are then divided into groups based on the magnitude of their association with CBCL syndrome scales using incrementally increasing *p*-value cut points (*p* ≤ 0.001, ≤ 0.01, ≤ 0.05, ≤ 0.1, ≤ 0.5, and ≤ 1.0). Polytranscript risk scores were calculated by multiplying the standardized expression levels (z-scores) for selected genes by the weight of their association to a phenotype (*t*-value), in this case a specific CBCL subscale, then computing the sum of weighted *z*-scores across selected genes. A 5-fold cross-validation approach was used to estimate the amount of variance in CBCL scales explained by our novel CBCL polytranscript risk scores: 80% of the probands were randomly selected to be used as a training set to derive gene weights for polytranscript risk scoring, which were then used to calculate polytranscript risk scores and analyze their association with CBCL scales in the remaining 20% of probands. Polytranscript risk scores were also calculated in our sample of 95 probands using transcriptome-wide blood-based meta/mega-analysis summary statistics for bipolar disorder (**BD**) (*n* cases = 95, *n* controls = 111)(8), schizophrenia (**SCZ**) (*n* cases = 285, *n* controls = 275)(9), major depression (**MDD**) (*n* cases = 744, *n* control = 646) (Hess et al., [unpublished]), and autism spectrum disorder (**ASD**) (*n* cases = 626, *n* controls = 447)(10). A nested logistic regression analysis was performed to estimate the proportion of variance in CBCL scales (set as response variable) explained by polytranscript scores after adjustment for covariates. The first model specified polytranscript risk scores as a predictor and sex, age, RNA quality, race, and SV1 as covariates. A second logistic regression was fit with polytranscript risk scores withheld from the model, thus the predicted probabilities for CBCL scales were dependent only on covariates. Variance in affection status explained by each model (Nagelkerke’s pseudo-*R*^2^) was calculated using the *R* package *fmsb* (version 0.6.3). We determined the variance in affection status explained by polytranscript risk scores by subtracting the *R^2^* estimates obtained from nested logistic regression models.

**References**

1. Liao Y, Smyth GK, Shi W (2014): FeatureCounts: An efficient general purpose program for assigning sequence reads to genomic features. *Bioinformatics*. 30: 923–930.

2. EMBL-EBI (n.d.): Gencode v19. . Retrieved from https://www.gencodegenes.org/releases/19.html.

3. Robinson MD, McCarthy DJ, Smyth GK (2009): edgeR: A Bioconductor package for differential expression analysis of digital gene expression data. *Bioinformatics*. 26: 139–140.

4. Fromer M, Roussos P, Sieberts SK, Johnson JS, Kavanagh DH, Perumal TM, *et al.* (2016): Gene expression elucidates functional impact of polygenic risk for schizophrenia. *Nat Neurosci*. 19: 1442–1453.

5. Langfelder P, Horvath S (2008): WGCNA: an R package for weighted correlation network analysis. *BMC Bioinformatics*. 9: 559.

6. Subramanian A, Tamayo P, Mootha VK, Mukherjee S, Ebert BL, Gillette M a, *et al.* (2005): Gene set enrichment analysis: a knowledge-based approach for interpreting genome-wide expression profiles. *Proc Natl Acad Sci U S A*. 102: 15545–50.

7. Purcell SM, Wray NR, Stone JL, Visscher PM, O’Donovan MC, Sullivan PF, Sklar P (2009): Common polygenic variation contributes to risk of schizophrenia and bipolar disorder. *Nature*, 2009/07/03. 460: 748–752.

8. Hess JL, Tylee DS, Barve R, de Jong S, Ophoff RA, Kumarasinghe N, *et al.* (2019): Transcriptomic abnormalities in peripheral blood in bipolar disorder, and discrimination of the major psychoses. *Schizophr Res*. . doi: 10.1016/j.schres.2019.07.036.

9. Hess JL, Tylee DS, Barve R, de Jong S, Ophoff RA, Kumarasinghe N, *et al.* (2016): Transcriptome-wide mega-analyses reveal joint dysregulation of immunologic genes and transcription regulators in brain and blood in schizophrenia. *Schizophr Res*. 176: 114–124.

10. Tylee DS, Hess JL, Quinn TP, Barve R, Huang H, Zhang-James Y, *et al.* (2017): Blood transcriptomic comparison of individuals with and without autism spectrum disorder: A combined-samples mega-analysis. *Am J Med Genet Part B Neuropsychiatr Genet*. 174: 181–201.
